# O04 Oral antimicrobial therapy for infective endocarditis: real-world experience from a teaching hospital in Scotland

**DOI:** 10.1093/jacamr/dlaf239.004

**Published:** 2026-01-05

**Authors:** Nikolas Rae, Emma Duncan, Neil Stevenson

**Affiliations:** Infection Unit, Ninewells Hospital and Medical School, Dundee, DD1 9SY, UK; Infection Unit, Ninewells Hospital and Medical School, Dundee, DD1 9SY, UK; Infection Unit, Ninewells Hospital and Medical School, Dundee, DD1 9SY, UK

## Abstract

**Background:**

Treatment of infective endocarditis with oral antibiotics is not common in the UK despite an evidence base supporting this approach in appropriate patients. From late 2022 onwards the Infection Unit in Ninewells Hospital, Dundee, has adopted the use of oral antibiotic therapy in the treatment of selected patients with infective endocarditis.

**Objectives:**

We aimed to review the clinical efficacy and outcomes in patients treated with oral antimicrobial therapy for infective endocarditis in our unit.

**Methods:**

We retrospectively reviewed patients treated with oral antimicrobial therapy for infective endocarditis. Patient records were reviewed for basic demographics, endocarditis characteristics and treatment (both IV and oral). Adverse outcomes (defined as all-cause mortality, unplanned cardiac surgery, recurrence of bacteraemia or embolic events) were assessed for within 6 months of completion of therapy.

**Results:**

In total 29 patients were identified, of which 21 (72%) were male. Mean age was 67 (range 36-94). Of the patients included, 20 were classed as ‘definite’ endocarditis by Duke-ISCVID 2023 criteria, with the remaining 9 meeting ‘possible’ Duke-ISCVID 2023 criteria. Structures involved included aortic valve (15 patients), mitral valve (11 patients), ICED (2 patients) and tricuspid valve (1 patient), with 9 of the 27 valve infections occurring in prosthetic valves. In total, 15 of 29 (52%) patients had moderate to severe valvular regurgitation, 5 (17%) patients had a vegetation of 10mm or greater and 4 patients underwent surgery during the course of their illness. With regards to microbiology, our cohort included 14 patients with *Streptococcus* spp., 12 patients with *Staphylococcus aureus* (all MSSA), 2 patients with *Enterococcus faecalis* and one patient with coagulase-negative *Staphylococcus* spp. Mean peak C-reactive protein was 163 mg/L (range 17–400) with mean C-reactive protein at transition to oral therapy of 21 mg/L (range 3–98). In total, 22 patients (76%) met the criteria used in the POET trial. Mean duration of IV therapy was 15 days (range 6–30). Mean duration of oral therapy was 26 days (range 14–42). Mean total duration of therapy was 41 days (range 28-56). The final oral antibiotic regimens used included linezolid in 19 patients (65%), amoxicillin in 5 patients (17%), co-trimoxazole in 3 patients (10%) and amoxicillin with rifampicin in 2 patients (7%). Change of oral agent due to adverse drug effects was required in 6 patients, all occurring in patients prescribed linezolid. Adverse outcomes occurred in three patients (embolic events in two patients and one death 4 months after treatment completion due to cardiac failure), with 5 patients yet to complete the full 6 month follow-up period.

**Conclusions:**

Oral antimicrobial therapy can be used in appropriate patients with infective endocarditis, with good efficacy and relatively low rates of adverse effects despite significant complexity of illness. Alteration of oral regimen was frequently required when linezolid was used.

